# Blood lead monitoring in a former mining area in Euskirchen, Germany: results of a representative random sample in 3- to 17-year-old children and minors

**DOI:** 10.1007/s11356-022-23632-2

**Published:** 2022-10-20

**Authors:** Jens Bertram, Christian Ramolla, André Esser, Thomas Schettgen, Nina Fohn, Jasmina Steib, Thomas Kraus

**Affiliations:** 1 Institute for Occupational, Social and Environmental Medicine, University Hospital Aachen, Pauwelsstr. 30, 52074 Aachen, Aachen, Germany; 2 Public Health Department Euskirchen, District of Euskirchen, Germany

**Keywords:** Children, Minors, BLL, Former, Lead, Mining

## Abstract

Heavy metal residues in former mining areas can pose a burden to the local environment and population even decades after closure of the mining sites. In the North Rhine-Westphalian (Germany) communities of Mechernich and Kall, both parts of the district of Euskirchen, lead residues are a source of health concerns for local residents. A statistically representative collective of both communities depending on sex, age, and area of residence was created, mirroring the local underage population. The blood lead levels (BLL) of 182 children and minors in the two adjacent communities were assessed via ICP-MSMS. The results were compared to German lead reference values, valid for the general underage population. In total, 32 (17.6%) of the subjects investigated exceeded the according reference values of 15 µg/L and 20 µg/L, respectively, depending on sex and age, thus pointing out an additional lead burden affecting children in the area. Potential lead sources contributing to the BLL were evaluated using a questionnaire. Factors that showed significant impact on the BLL were, other than age, sex, height, and weight, the factors occupancy, time spend in the garden, garden hand-to-mouth contact, consumption frequency of homegrown products, and lifestyle factors. The data presented enable both residents and the local authorities to further reduce lead exposure and to take appropriate personal and public action.

**Supplementary Information:**

The online version contains supplementary material available at 10.1007/s11356-022-23632-2.

## Introduction

Lead is known to cause a variety of adverse health effects. Among the most concerning effects on children is the negative impact on the development of cognitive functions. The damage caused by early and long-term lead exposure is difficult to determine, but effects on the development of cognitive functions have been reported to occur already below blood lead concentrations of 100 µg/L or even 50 µg/L. Potential consequences might be a delayed reading readiness (McLaine et al. 2013) or a lower intelligence quotient (Min et al. 2009) and an increased risk of developing attention deficit hyperactivity syndrome (ADHS) (Human Biomonitoring Commission (HBM Commission) 2009; Lanphear et al. 2000). Lead exposure during pregnancy might result in reduced birthweight. Since lead is known to pass the placental barrier, the BLL of the mother are associated with that of the child. Early life lead exposure also is inversely correlated with growth. Delayed puberty was also observed in girls and with lesser certainty in boys (Agency for Toxic Substances and Disease Registry (ATSDR) 2020). Various other systems and functions of the human body are affected from high and repeated lead exposure of several hundred µg lead/L blood as well, including the hematological (inhibition of enzymes involved in the heme synthesis like delta-aminolevulinic acid dehydratase (δ-ALAD)), renal (proteinuria, interstitial and peritubular fibrosis), cardiovascular (increased systolic and diastolic blood pressure), and reproductive systems, and have been described thoroughly in the literature. In contrast to other adverse and toxic effects, the mechanisms behind the disturbance of the hemoglobin synthesis are largely understood. After lead is absorbed into the bloodstream, it can bind to proteins which then inhibit the beforementioned enzymes related to the heme synthesis and thus the hemoglobin synthesis. Effects on the gene level related to the polymorphic nature of δ-ALAD and lead exposure are also discussed (Qader et al. 2021).

Fortunately, these are phenomena, in general occurring with BLLs in the abovementioned high concentrations related to an occupational environment (International Agency for Research on Cancer (IARC) 2006; Permanent Senate Commission for the Investigation of Health Hazards of Chemical Compounds in the Work Area (MAK-Commission) 2019).

However, scientific evaluation has not come to a standstill. For instance, lead has been categorized as a category 4 hazardous substance (that is, with a non-genotoxic mechanism) by the German Research Foundation (DFG) (2021) (after classification as category 2 substance, that is, carcinogenic to animals since 2007 (DFG 2007)). To date, the IARC (2006) classified inorganic lead as a category 2A substance, that is, as probably carcinogenic to humans. According to the current state of scientific knowledge, there is no lead concentration that can be considered as harmless (HBM Commission 2009).

Formerly, the use of lead in paints and water pipes as well as in anti-knock agents formed the biggest health threats to the general population and the environment. Typical lead sources to date are solder materials, ammunition, and batteries (Bae et al. 2020; Lelievre et al. 2020; Pohl et al. 2017). Contaminated soils in the vicinity of active or former mines, smelters, or recycling sites pose a permanent source of lead, contributing to elevated blood lead levels compared to the general population (Childebayeva et al. 2021; Jo et al. 2021; Shi et al. 2021).

Since the ban and phasing out of leaded fuel as the major source of lead exposure in the European Union and worldwide from the 1970s on, BLL declined over decades in the general population (Egan et al. 2021; Ettinger et al. 2020; Lermen et al. 2021; Smolders et al. 2010; von Storch et al.^2003^). Other measures, for example, in the United States, focusing on the reduction of lead residues in old coatings of buildings and the amount of lead allowed in children’s products, have been undertaken or are being implemented (President´s Task Force in Environmental Health Risks and Safety Risks to Children 2018). The improved situation resulted in the need to update existing reference values (RV) for the yet lower exposed general population (Center for Disease Control (CDC) 2018). Existing German reference values were revised in the last decade for males, females, and ultimately for children with new data from the German Environment Agency (UBA) (2019), HBM Commission (2009, 2019), and Vogel et al. (2021). The revised German biological reference values for children and minors in Germany are the following:

- 3- to 10-year-old males: 20 µg/L
- 11- to 17-year-old males and 3- to 17-year-old females: 15 µg/L

However, lead residues remain in the area of old mines, thus posing an environmental long-time burden. Lead ore from the region discussed here was extracted for centuries, and the spot was considered as one of the biggest lead ore deposits in Europe, affecting the surrounding communities of Mechernich and Kall (district of Euskirchen). In 1957, mining activities came to an end due to economic reasons. Soil samples from land designated as building ground, analyzed in the region, were reported to show median lead concentrations of 4380 mg/kg, thus exceeding the maximum allowed lead concentration in soil for real estate (400 mg/kg) by the factor of 10 (Günther et al. 2020). In areas adjacent to the river Bleibach (“lead river”) and widespread hotspots, lead soil concentrations of more than 10,000 mg/kg have been reported, covering more than half of the area discussed (Schalich et al. 1986).

The effects of lead-contaminated soils or emissions from the lead treating industry on BLL are also described in the literature (Childebayeva et al 2021; Jo et al. 2021; Mattisson et al. 2020). Remediation measures were partially implemented in order to meet the concern of local residents. However, remediation of lead contamination is reported to sometimes require harsh steps, among others, exchanging and covering of contaminated soils, and might therefore be costly and challenging (Chowdhury et al., 2021).

Seasonal changes have to be considered when planning a biomonitoring study. They have been reported to have an effect on the BLL. During the warm period, elevated BLL were observed in comparison to cooler seasons (Laidlaw et al. 2016; Zahran et al. 2013).

The main pathways for lead absorption are the oral and the inhalation pathways, the former varying strongly with age. Adults absorb 10% of the ingested lead, while children absorb up to 50% which is critical especially when it comes to hand-to-mouth contact with contaminated soils (DFG 2000). As known from the literature, lead binds to hemoglobin and is excreted via urine and feces. With a blood lead half-life time of about 30 days, the BLL mainly resemble the exposure of the past few months and therefore is routinely used for biomonitoring. No BLL that can be considered as safe exist (ATSDR 2020).

In a former study, conducted in July 2019 in the area of Kall and Mechernich (Bertram et al. 2022), the blood of 506 volunteers comprising all age classes from children to senior citizens was analyzed for lead. No difference in the BLL compared to the general population was observed in the participating adult population. In contrast, hints for a higher than assumed number of children and minors exceeding the background burden of the general population or the 95th percentile from the German Environment Agency were observed (UBA 2019). However, with only 36 underage volunteers participating, these data lacked statistical robustness. The paper at hand describes the follow-up investigation in children and minors undertaken in order to clarify a potential exceedance in contrast to the background burden of the general population. A statistical representative sample of children and minors living in the region was created, and contributing factors were assessed via a questionnaire. Since a statistical representative sample takes into account all kind of factors that might jeopardize the outcome of the analysis, it is considered the gold standard for studies that intend to clarify the background of the general population. Biasing factors like differences of the soil depending on the region, effects of age and sex, dietary issues differing between families, or prevailing smoking habits thus should be taken into account by statistical representative sampling. Measures to reduce BLL are discussed.

## Methods

### Sampling time, sample collective, and sample collection

Blood sampling was performed a week before the summer holidays at the end of June 2021 in order not to underestimate the BLLs. Participants were selected randomly. A sample size of 200 participants was calculated as necessary to determine deviations from the background burden of the general population with a strong statistical power (see also statistical methods). In order to determine the influence of various factors on the BLL, various potential sources of lead were evaluated using a questionnaire. The sample collective selected was aimed to match the fractions of the populations’ distribution according to sex, age, and community of residence. The proportion of candidates was rounded to full numbers, resulting in 206 potential participants, contacted initially.

Inclusion criteria were residence for at least 4 months in one of the two communities and the desired age between 3 and 17 years. Exclusion criteria were pregnancy or symptoms of a COVID-19 disease or contact with a COVID-19–infected person, respectively.

Participants were selected randomly from data provided by the local medical authority using an Excel random function until the desired number of participants for sex, age, and community was reached in the first recruitment phase. Chosen potential candidates themselves (if 16 years or older), or their legal guards, were contacted in a first step by mail and informed briefly about the project and asked about their willingness to participate. In case of a positive response, the participants or their legal guards received the full information material, a declaration of consent, a privacy note, and a questionnaire. Teenagers 16 years of age or older were able to give their consent themselves.

The sampling was performed on 4 consecutive days at the end of June 2021 with an extra appointment 2 weeks later for people missing their initial appointments. Samples were stored in 4.9 mL EDTA-monovettes (Sarstedt, Nümbrecht, Germany) in a refrigerator at + 4 °C overnight and brought to the laboratory the next morning. Samples were stored at − 20 °C until analysis.

**Fig. 1:**
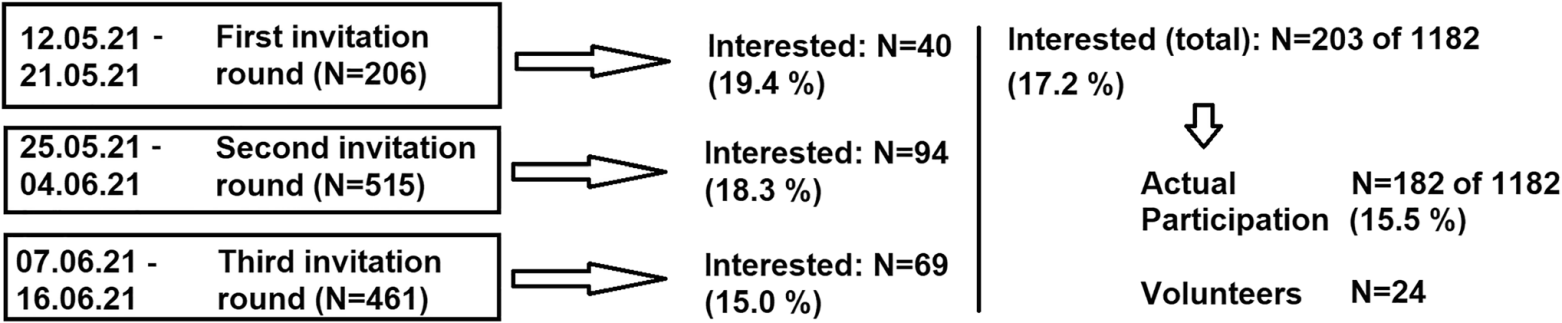
Recruitment scheme of the sample collective

Unfortunately, about 80% of the potential participants or legal representatives did not signal interest in participating in the study after the initial recruitment phase; thus, more recruitment letters were distributed in two additional recruitment phases, contacting about five times as many people as needed to match the non-responders (see Fig. 1). The sampling in the two additional recruitment phases was randomized as well. Only potential participants who could fill the gaps between the targeted participants and the actual participants were invited. The exact constitution of the sample collective can be found in the supplemental materials.

### Laboratory analysis

Two hundred µL of blood sample was diluted 1:50 with Millipore water, 20-µL 65% HNO_3_, and 300-µL Triton X 10%. One hundred µL rhodium 1 mg/L was added as internal standard (Merck, Darmstadt). The final volume was 10 mL. The calibration standards ranged from 20 to 7000 ng/L and comprised 9 doted blood samples as standards including a blank.

Samples were centrifuged for 10 min at 3000 rpm, the sedimented residue was discarded, and the liquid samples were measured on an ICP-MSMS Agilent 8900 (Agilent Technologies, Waldbronn, Germany) on mass 207 in the SQ-He Mode, using Rh 103 in the same mode, as internal standard. The limit of quantification (LOQ) of the method was 0.1 µg/L and therefore sufficiently low for environmental sample analysis. The laboratory successfully participates in international round-robins of the German External Quality Assessment Scheme (G-EQUAS) of the Friedrich-Alexander University of Erlangen-Nuremberg. Control materials were measured with each analytical run as a quality control. Two controls were measured every 10 samples. After 5 samples, a sample blank was measured.

### Questionnaire

The participants or their legal representatives were asked to fill out a questionnaire covering sex, age, height, weight, birthplace, residence, occupancy, availability of a garden space and time spent in the garden, playing habits, diet, smoking and passive smoking as well as other lifestyle factors like hunting or shooting, renovation works, or other, thus covering the common confounders reported in similar studies (Lermen et al. 2021; Mattisson et al. 2020). The complete questionnaire can be viewed in the “Supplementary information” section.

### Statistics

An a priori calculation of the sample size necessary indicated that 200 subjects were required to achieve a statistical power of 80% at a significance level of 0.05 and a required doubling of the odds ratio.

To determine whether the number of exceedances of individual reference values was significantly increased in the study cohort compared with the corresponding general population, the Agresti-Coull confidence interval was calculated for the prevalence of exceedances (Brown et al. 2001). Due to the nature of biological reference values, 5% exceedances of these values must always be expected in a population. Therefore, the abovementioned method is used to determine the number of exceedances above which there is a significant difference to the comparison cohort, in this case the general population. The calculations showed that from the 17th exceedance found, there were significantly more exceedances in the study cohort than in the corresponding general population.

Differences in groups of covariates were examined by univariate ANOVA. Correlation analyses were conducted as non-parametric correlation for categorical values and as Spearman correlation for parametric variables.

All analyses were conducted with the program SPSS (IBM Statistics SPSS 25).

## Results

### Representative sample collective

After three invitation rounds, 203 people consented to participate in the study, of whom 184 eventually participated. Two of the participants did not consent data storage and processing and therefore received only their medical report. These were excluded from further data analysis. In addition to the remaining 182 participants blood samples, another 24 samples of volunteers were collected (see Fig. 1). The volunteers were not included in the data analysis as well.

As visible in the “Supplementary information” section, the local population was matched in a satisfying manner. However, the response of participants in Kall was lower than aimed at (78.3%) and comparatively lower than in Mechernich (92.5%), but the overall distribution given the poor willingness to participate in the study was satisfying. Around 48.5% of the children and minors living in Mechernich and Kall were female and 51.5% male. Of the 182 participants eventually participating, 83 (45.6%) were of female and 99 (54.4%) of male sex. The actual percentage of participants aimed at depending on the age group varied between 75 and 108% with lower participations for the 3-year-old (43%) and the 16-year-old (64%).

### Results from the questionnaire

Height and weight as well as BMI showed no abnormalities. Occupancies of the tested participants were normally distributed. All but 8 subjects lived in the region for 3 years or longer. The year of construction of the houses in question was evenly distributed across the sample collective. The majority of the participants was able to use a garden that belonged to the domicile. The time spent in the garden decreased from 3- to 10-year-olds to 11- to 17-year-olds, as well as the time spent outside as can be seen from the mean data in Table 1. Hand-to-mouth contact was reported 5 times (6.0%) in children aged 3 to 10 years. None of the participants had access to an own fresh water well. Parental smoking in front of the children was rare (*n* = 10 or 5.5%) as well as the number of minors (*n* = 5 or 2.7%) that reported smoking. Lifestyle exposure, like dealing with old paint during renovation works or shooting in a gun club, was reported in 11 cases (6.0%), even in children aged 3 to 10 years. Lifestyle exposure was reported with contact to paints during renovation work and in one case because of membership in a shooting club. Three cases of anemia were reported in 11- to 17-year-old females. Pre-existing conditions were reported in 46 cases (25.3%) with greater abundance in older children (see Table 1). Among the great variety of mostly allergic pre-existing conditions, three of the children were reported to suffer from attention deficit hyperactivity disorder (ADHS) or autism. All three exceeded the according RVs. Medicament consumption was frequent with *n* = 42 (23%) of the subjects taking pharmaceutical drugs but did not correlate with BLL.

**Table 1:** General data from the questionnaire presented in the 4 subgroups depending on age and sex. n.s., not specified

			Male 3–10	Female 3–10	Male 11–17	Female 11–17
N			50	39	49	44
Age [a]			6.6 (3–10)	6.8 (3–10)	14.1 (11–17)	13.9 (11–17)
Residence	Kall		12	11	11	13
	Mechernich		38	28	38	31
Height [cm]			124.8 (100–159)	125.3 (98–158)	171.5 (144–196)	164.7 (148–178)
Weight [kg]			25.3 (15–43)	25.5 (14–44)	62.7 (34–100)	54.9 (35–86)
BMI [kg/m^2^]			16.0 (13–21)	15.4 (0–20)	20.8 (0–37)	19.0 (0–30)
Occupancy [a]			5.4 (1–10)	5.6 (2–10)	11.6 (3–17)	11.8 (1–17)
Construction year of residence	< 1973		15	18	14	14
	1973–2000		9	4	13	12
	> 2000		24	13	22	16
	n.s		2	4		2
Garden	Yes		46	37	49	40
	No		4	2		3
	n.s					1
Garden time [h/w]			14.7 (2–37.5)	11.5 (0–35)	7.7 (0–45.00)	9.9 (0–25)
Garden hand-to-mouth contact	Yes		3	2		0
	No		47	34	48	44
	n.s			3	1	
Time spent outside [h/w]			6.6 (0–30)	6.0 (0–60)	3.1 (0–15)	2.0 (0–10)
Consumption of homegrown products	Daily			1	1	2
	Several times a week	11	12	8	5	
	Once a week	4	2	1	7	
	Seldom		13	12	19	10
	Never		22	10	19	20
	n.s			2	1	
Fresh water well	Yes					
	No		49	39	49	44
	n.s		1			
Consumption of bowels	Yes		1	3	4	1
	No		48	36	45	43
	n.s		1			
Parental smoking in front of children	Yes	Daily			2	4
		Several times a week	1			
		Once a week				
		Seldom	1		1	1
	No		48	39	46	39
Amount per day			8.5 (7–10)	11 (5–18)	13 (3–20)	11.4 (2–20)
Smoker	Yes	Daily				
		Several times a week			2	
		Once a week				1
		Seldom			1	1
	No		50	39	45	42
	n.s				1	
Amount per day					n.s	n.s
Lifestyle exposure	Yes		3	2	2	4
	No		10	4	7	5
	n.s		37	33	40	35
Anemia	Yes					3
	No		50	39	49	41
Pre-existing conditions	Yes		7	5	17	17
	No		42	33	32	27
	n.s		1	1		
Drugs/medicaments	Yes		8	4	16	14
	No		41	34	33	29
	n.s		1	1		1

### BLL

All BLL were above the LOQ. From the 182 blood samples investigated, 32 (17.6%) were above the according reference values from the German Environment Agency depending on sex and age with a maximum value of 44.0 µg/L lead in the blood. In the community of Mechernich, 27 (20.0%) out of 135 participants had BLL above the reference values, while in Kall, only 5 (10.6%) out of 47 participants were above the reference values.

Of the 24 extra blood samples collected, another 5 (20.8%) exceeded the according reference values (data not shown).

Descriptive statistics are given in Table 2.

**Table 2:** Descriptive statistics of the BLL in µg/L of the 182 selected study participants in the communities of Mechernich and Kall in comparison with the German reference values (RV) (UBA 2019). n.s., not specified

						Lead [µg/L]						
Residence sex and age	*N*	Min	Max	Mean	Median	5. Perc	1. Quartile	3. Quartile	95. Perc	RV	> RV	> RV [%]
Mechernich												
Mechernich male 3–10	38	5.8	37.6	13.6	12.6	6.0	8.2	16.1	29.9	20	6	15.8
Mechernich male 11–17	38	4.4	40.9	13.1	10.4	5.7	7.5	17.1	33.7	15	12	31.6
Mechernich female 3–10	28	4.9	37.4	13.1	11.0	5.0	8.3	16.2	31.3	15	8	28.6
Mechernich female 11–17	31	4.6	17.2	8.2	7.4	4.9	5.8	9.9	15.7	15	1	3.2
Mechernich female	59	4.6	37.4	10.5	8.6	5.1	6.8	12.6	23.7	15	9	15.3
Mechernich male 11–17 and female	97	4.4	40.9	11.5	9.6	5.1	7.1	14.5	24.0	15	21	21.6
Mechernich total	135	4.4	40.9	12.1	10.2	5.6	7.3	14.7	24.9	–	27	20.0
Kall												
Kall male 3–10	12	7.3	44.0	16.2	13.3	7.3	9.5	16.5	n.s.	20	2	16.7
Kall male 11–17	11	5.3	31.4	10.6	9.3	5.3	6.1	11.5	n.s.	15	1	9.1
Kall female 3–10	11	4.5	35.0	13.0	12.1	4.5	9.1	13.6	n.s.	15	1	9.1
Kall female 11–17	13	3.9	15.2	8.2	7.5	3.9	6.4	9.7	n.s.	15	1	7.7
Kall female	24	3.9	35.0	10.4	9.4	4.1	6.5	12.3	30.1	15	2	8.3
Kall male 11–17 and female	35	3.9	35.0	10.5	9.3	4.4	6.3	12.0	32.1	15	3	8.6
Kall total	47	3.9	44.0	11.9	10.3	4.8	7.3	13.0	33.6	–	5	10.6
Total male 3–10	50	5.8	44.0	14.2	12.7	6.3	8.9	16.1	33.1	20	8	16.0
Total male 11–17 and female	132	5.1	40.9	11.2	9.6	5.1	7.1	12.8	24.3	15	24	18.2
Total	182	3.9	44.0	12.1	10.2	5.6	7.3	14.5	28.5	–	32	17.6

**Fig. 2:**
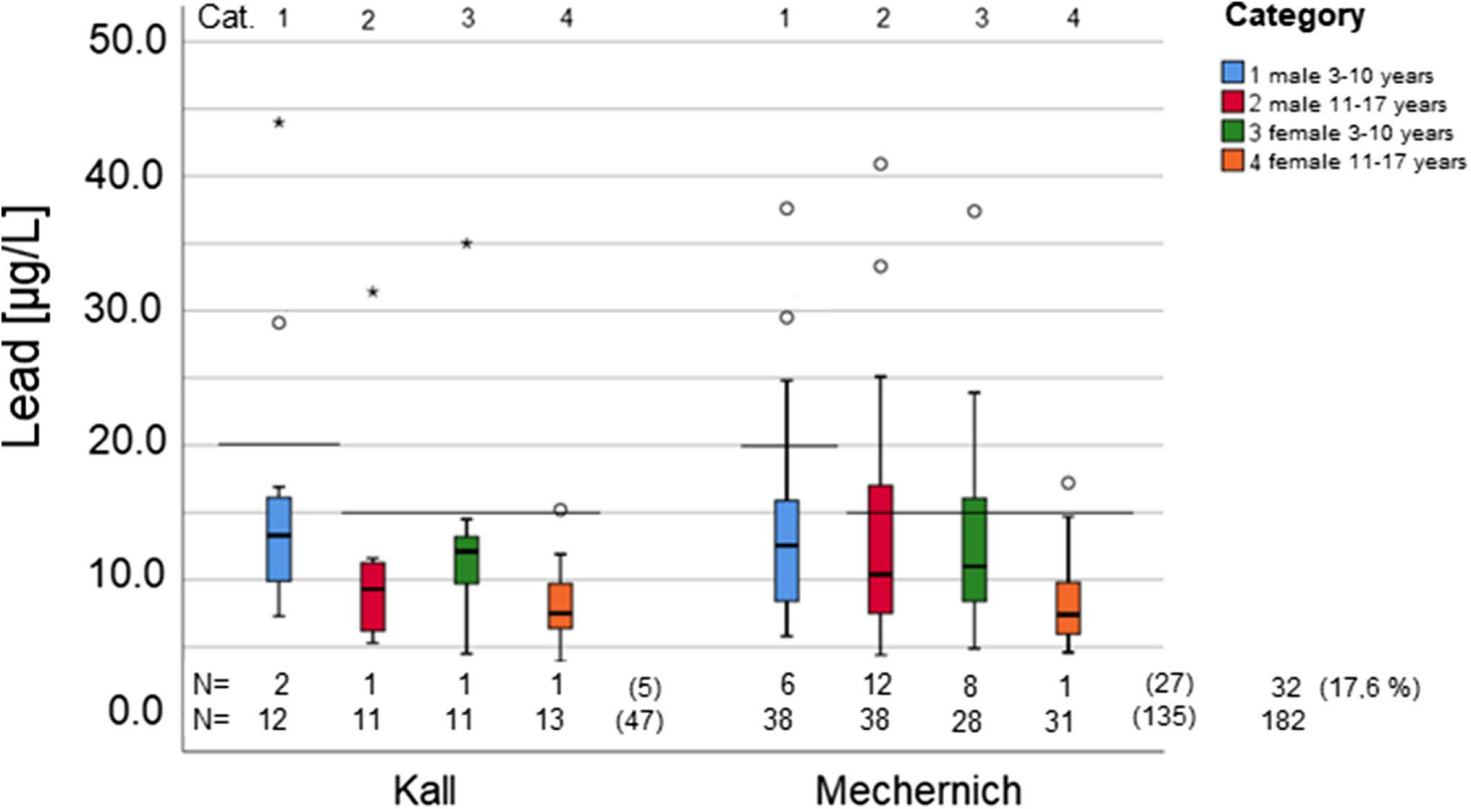
BLL in the two communities depending on sex and age and according reference values; Cat. 1, males 3 to 10 years; Cat. 2, males 11 to 17 years; Cat. 3, females 3 to 10 years; Cat. 4, females 11 to 17 years. Reference values: 15 µg/L and 20 µg/L indicated for according categories

The results, categorized in four groups, for age and sex for each community, are depicted as boxplots in Fig. 2 with the according RVs.

**Fig. 3:**
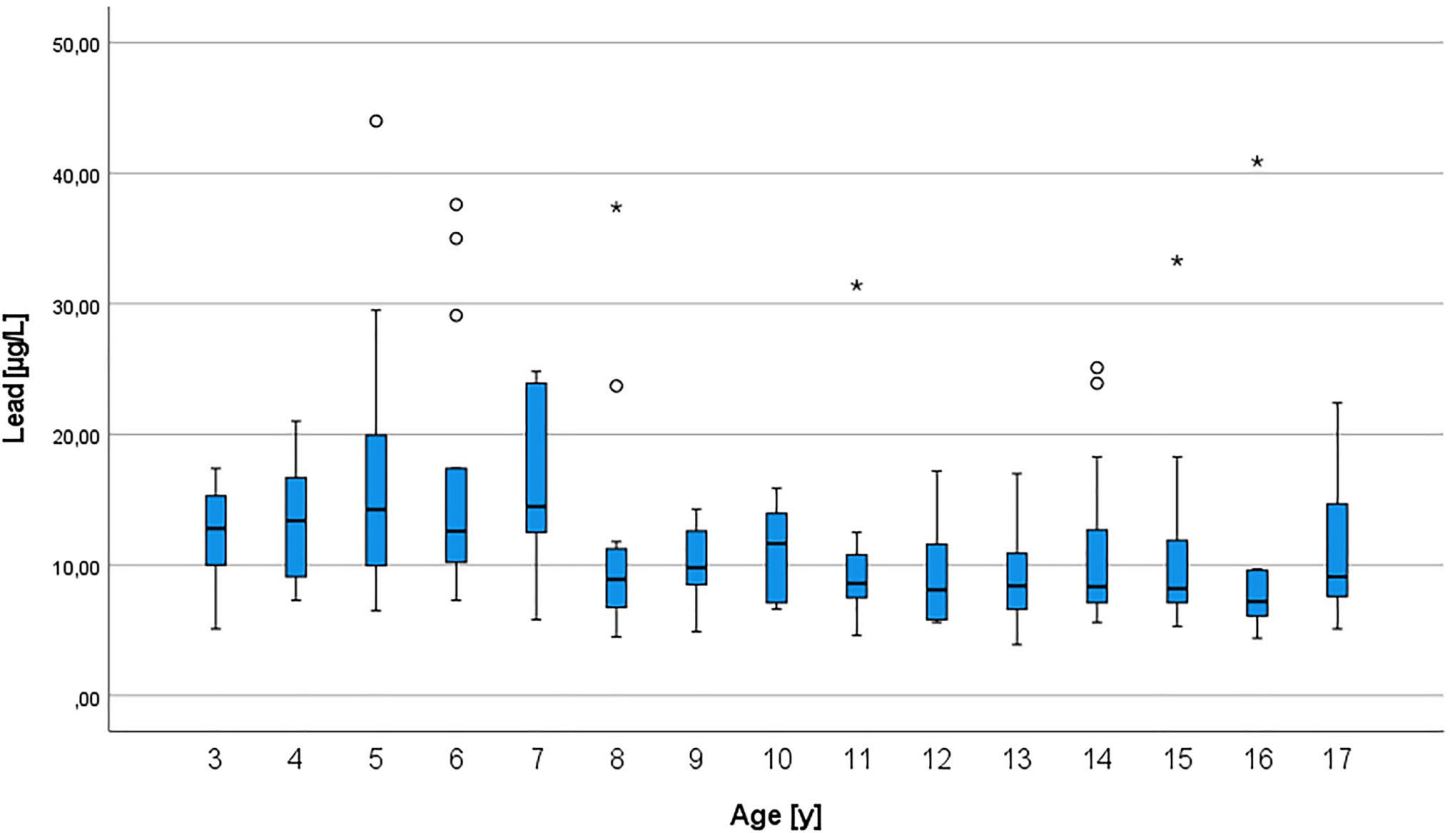
BLL depending on age per year

For a better resolved display of the monitored BLLs, the BLLs are depicted by BLL per year in Fig. 3.

Age, weight, height, occupancy, garden time, and time spent outside were significantly correlated to the BLL (see Table 3). All but garden time and time spent outside were negatively correlated with the BLL.

**Table 3:**
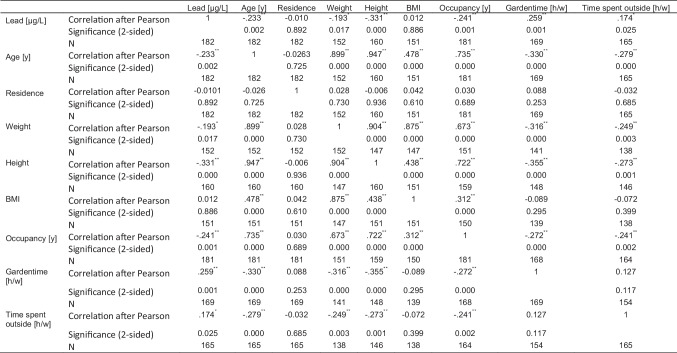
Correlation table

^**^The correlation is significant on the 0.01 level (2-sided). *The correlation is significant on the 0.05 level (2-sided)

UNIANOVA demonstrated significant effects of four variables on the BLL. The BLL were demonstrated to be significantly higher in males than in females (*p* < 0.05) (data not shown). Garden hand-to-mouth contact was stated 5 times with yes, and BLL resulted to be higher in those subjects that had hand-to-mouth contact in the garden than in those who had not (*p* < 0.05).

**Fig. 4:**
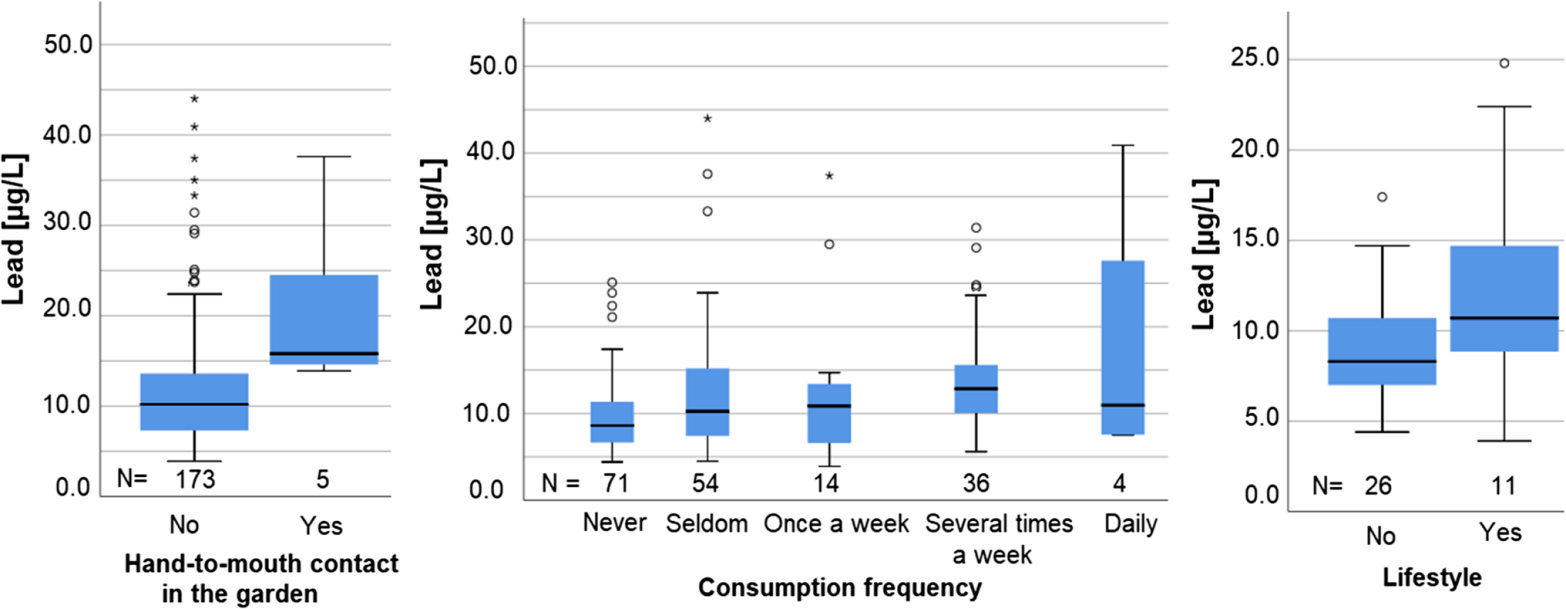
Boxplots of BLL and garden hand-to-mouth contact, consumption frequency of homegrown products, and lifestyle

The consumption-frequency of homegrown fruits and vegetables was significant with *p* < 0.05 resulting in a higher BLL with more frequent ingestion, as well as lifestyle factors (*p* < 0.05), the latter with the restriction of only 37 completed questionnaires for this question. Boxplots are depicted in Fig. 4. The homegrown products most frequently consumed were strawberries (*N* = 43), raspberries (*N* = 34), tomatoes (*N* = 25), cherries (*N* = 24), salad (*N* = 21), apples (*N* = 20), cucumbers (*N* = 20), currants (*N* = 13), and carrots (*N* = 11).

**Fig. 5:**
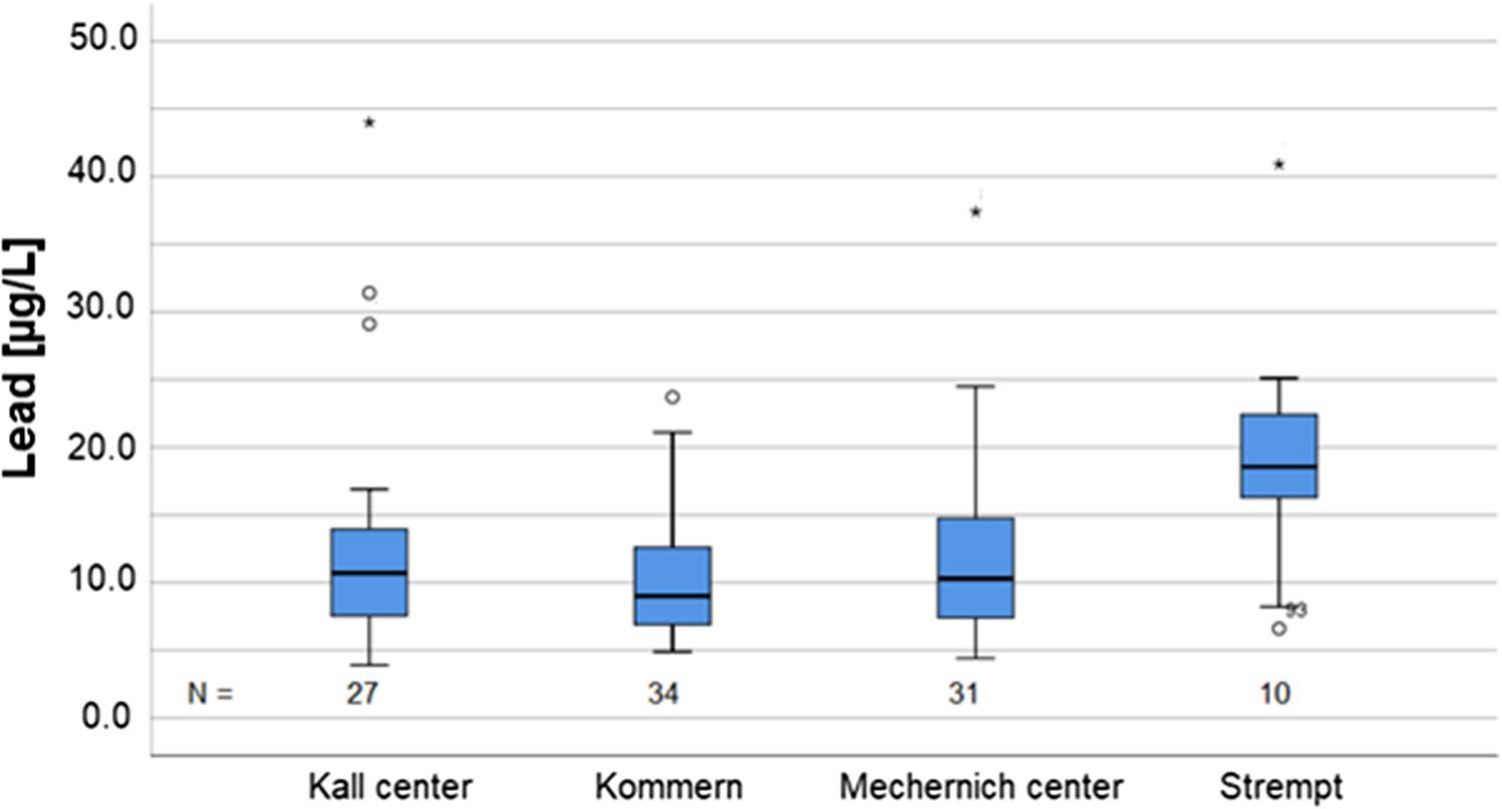
BLL distribution in the major localities of Kall center, Kommern, Mechernich center, and Strempt

Regarding the localities within the communities with the highest number of participants in more detail, (namely, Kall center, Mechernich center, Mechernich-Kommern, and Mechernich-Strempt), exceedances of the RVs in more than 5% of the cases could be observed. This additional information might help the local authorities to establish a priority list for exposure reduction measures. Given the smaller number of children per locality, all but Strempt lacked statistical robustness but were nevertheless significantly different from the background burden of the general population. Of the most populated localities, Mechernich-Strempt showed significantly higher lead values than the rest. UNIANOVA proved a significant difference among these 4 localities with a *p* value of < 0.05. Mean BLLs were 12.9. µg/L for Kall-center, 12.0. µg/L for Mechernich center, 12.4 µg/L for Kommern, and 19.5 µg/L for Strempt (refer to Fig. 5).

In Kall center, 4 (14.8%) out of 27 subjects had BLL above the RVs. In Mechernich center a number of 6 (19%), in Mechernich-Kommern a number of 5 (15%), and in Mechernich-Strempt a number of 7 (70%) BLL exceedances of the according RV were observed. That is, 22 (21.6%) of 102 subjects investigated in these four localities had BLL above the RV. One of the exceedances can be explained by a membership in a gun club. The remaining 10 RV exceedances in 80 subjects equaled 12.5% of the residual subjects.

## Discussion

### Sampling time and sample collective

The study was conducted in June 2021, before the summer holidays. This was supposed to maximize the BLLs. On the one hand, the likelihood of inhaling residual dusts is greatest in the summer months due to the dry, warm climate. On the other hand, the likelihood of consuming lead-contaminated fruit and vegetables from home cultivation is also high during this time, since many fruits and vegetables are ready for harvest at this time. In addition, the summer months are the months in the year where most of the time is spent outside due to the temperatures and the long daylight phases. This also maximizes the possibility of coming into contact with potential lead-containing materials (Laidlaw et al. 2016; Zahran et al. 2013). A sampling time before the summer holidays was given preference over a sampling time during or after the summer holidays. As stated in the “Introduction” section, the biological half-life time of lead in the blood is approximately 30 days and reflects the actual lead exposure of the last weeks and months (MAK-Commission 2019). Therefore, sampling during or after the summer holidays might not have reflected on-site exposure, but a mixed exposure resulting from on-site exposure and any holiday location outside the region. Despite of a lockdown with closed schools and kindergartens, contact limitations due to the COVID-19 pandemic, and a rather cool May in 2021, the sampling is considered as realized under normal and representative conditions. The children’s behavior to play outside should not have been affected due to the closure periods of schools and kindergartens nor due to reduced contacts caused by federal contact restrictions along the COVID-19 pandemic.

The overall desire to participate in the study reflected in the response rate was poor. As reported by Galea and Tracy (2007), participation rates in epidemiological studies decreased strongly from about 70% in the late 1970s to about 50% at the beginning of the new millennium. A wide range of motivations explaining the low participation rates could be discussed but would go beyond the scope of the study. It can be speculated that many of the residents invited already felt themselves well informed after a previous study conducted in volunteers of all age groups in 2019, evidencing no blood lead burdens that gave cause for concern in adults.

The amount and distribution of participants fell short under the desired number of participants but remained satisfying. Volunteers were excluded from the study to avoid contorting effects. The existing population distribution among minors from 3 to 17 years was matched very well with smaller downstrokes for the 3- and 16-year-olds. The study results are robust as visible from the strong statistical power of 99.9%.

### Human biomonitoring results in comparison to other studies

With 32 (17.6%) of the BLLs above the respective RV, the 5% threshold is clearly exceeded. As can be seen in Table 2, the BLLs of each of the subgroups analyzed in the district of Euskirchen are above the background burden of the general population.

In the first study conducted by the authors in June 2019, the number of minor participants was insufficient for statistically robust results. Six out of 36 children (16.7%) between 3 and 17 years exceeded the according RVs (Bertram et al. 2022). With a proportion of 17.6% RV exceedances, the study at hand confirms the ratio of RV exceedances observed initially.

The results of another investigation in the region by Einbrodt in 1985, which was unfortunately not published internationally, showed about tenfold higher BLLs, which was back in the 1980s common mainly due to the use of leaded gasoline. Einbrodt concluded that local hot spots must exist, which were also particularly noticeable in the region of Strempt. More to the region of Strempt further below.

As discussed in Bertram et al., overall BLLs in the region are low or equally low compared to other former mining sites worldwide (Bertram et al. 2022). For instance, Schoof et al. (2016) reported a decline of mean BLL in 1- to 5-year-old children in the former copper mining site of Butte in the United States after remediation measures from 34.8 to 15.3 µg/L, while BLLs remained high in a similar Australian location (Boreland et al. 2008). BLLs in the lead mine of Broken Hill decreased after remediation from 163 to 83 µg/L. In the mining town of Kabwe in Zambia, more than 95% of the children investigated had BLL above 100 µg/L (Bose-O'Reilly et al. 2018).

The authors speculate whether a more humid climate might, apart from flooding events, results in lower lead exposure due to precipitation of dusts or binding processes in the vegetation. If dryer and hotter and thus dustier summer months were to come in the next decades as a consequence of global climate change, these might precipitate in again increasing BLLs of the locals. More research is necessary to understand the underlying factors.

Vogel et al. (2021) presented data from the German Environmental Survey (GERES V 2014–2017). The categories presented there do not match the categories for the derived RVs by the German Environment Agency. However, the core statement remains the same. BLLs in Mechernich and Kall differ from the general German population. A summary of the most important data from Vogel et al. and our data is added in the “Supplementary information” section.

Vogel et al. (2021) also summarized human biomonitoring data for children from various countries like Belgium, Sweden, and Korea (Almerud et al. 2021; Burm et al. 2016; De Craemer et al. 2017), which unfortunately are difficult to compare to the study at hand. However, where applicable, mean results of these studies show lower BLLs as those found in Mechernich and Kall.

### Evaluation of the questionnaires

Age was negatively correlated with lead, which is in concordance of the higher German RV for 3- to 10-year-old males. BLLs were significantly decreasing with age. As shown in Fig. 3, mean BLLs per year were higher in 3- to 7-year-old children compared to the older ones. It is known from the literature that younger children absorb more lead after oral ingestion than adults (DFG 2000). On the one hand, the hand-to-mouth contact is more frequent in small children of this age. On the other hand, older children and minors value personal hygiene more, i.e., washing their hands more frequently, and show a reduced behavior to put objects in their mouths, so that the oral intake of lead-contaminated dust or even soil is reduced. It remains unclear whether the sharp decrease in BLLs that we observed between the 7- and 8-year-olds is due to biological transformations or behavioral changes.

Due to the fact that 90% of the lead binds to the membrane of erythrocytes, higher BLLs in male participants were to be expected (MAK-commission 2019). We found higher BLLs in males (mean BLL 13.4 µg/L) than in females (mean BLL 10.5 µg/L) which is visibly more pronounced than in the data from Vogel (mean BLL males: 10.9 µg/L, mean BLL females: 10.4 µg/L). The German RVs for adults are accordingly higher for males (40 µg/L) as for females (30 µg/L) (UBA 2019). These results are backed by international studies for children as well (Almerud et al. 2021; Burm et al. 2016; Health Canada 2019) and can be explained with the higher amount of red blood cells in males compared to females. A closer look at the four localities within the communities with the most participants reveals that they were all significantly different from the general population as well, but with a low statistical power, apart from Mechernich-Strempt which stood out with 70% of the 10 children and minors monitored, exceeding the RVs. This was also mentioned by Einbrodt in his unpublished report from 1982.

The residents of Mechernich-Strempt might be exposed to a more sever lead contamination of the adjacent river Bleibach. Lead soil concentrations above 10,000 mg lead/kg soil have been reported in 1986 in areas near the river Bleibach and potentially peaking in the area of Strempt (Schalich et al. 1986). Since these data lack actuality and resolution, new lead soil measurements should be initiated in the region. Above all, a large-scale disastrous flooding event took place in the region shortly after the conduction of the study. The big picture might have changed significantly due to soil and thus lead transfer processes related to the flooding; therefore, more recent soil data is required.

As already discussed in relation to age, hand-to-mouth contact contributes to the lead burden. Hand-to-mouth contact in the garden was asked about in the questionnaire because it’s likely to have the greatest impact on the BLL. It was rare but resulted in significantly higher BLL with up to 37.6 µg/L. Three 5-year-old children, one 6-year-old child, and one 7-year-old child were reported to have hand-to-mouth contact in the garden of whom 3 were boys and two were girls. The number of caregivers of younger children that reported hand-to-mouth contact in the garden seems astonishingly low. But the awareness of the caregivers for predominating lead contaminations in soils of the region and thus the gardens might result in a more pronounced protective behavior of the adults, hindering supervised children to excessively put objects or hands in their mouths, at least while playing in the garden as the assumed main source of lead.

The consumption frequency of homegrown fruits and vegetables contributed to the BLL as well, the more frequent they were consumed. Participants that did not consume homegrown products had lower BLL as those who did so. This can be explained either with residues of soil still present on the foods when ingested or soil lead that was incorporated into the plant. Lead can be integrated into different parts of the plants depending on the plant. Thus, different concentrations of lead can be found in the roots, the stem, the leaves, the fruit, or the skin (Finster et al. 2004; Hiller et al. 2022; Norton et al. 2015; von Hoffen and Saumel 2014). The majority of the homegrown products consumed were low-hanging fruits or vegetables with either direct soil contact or prone to dust precipitation.

The data received from the questionnaire was not sufficient to draw conclusions whether certain types of fruits and vegetables significantly could be held responsible to cause higher BLLs than others. However, the total consumption of homegrown foods as well as the consumption of strawberries and tomatoes correlated with the BLL. More careful cleaning of homegrown foods while avoiding ground-level vegetables and fruits, like potatoes and strawberries, is advisable in order to reduce BLL, in those cases where the respective RV was exceeded. Pealing vegetables that grow into the soil itself should be considered as well.

Regarding the lifestyle factors in more detail, especially the membership in a gun club and exposure to lead-containing paints during renovation works are known sources of lead and proved to be significant sources of lead in our study as well (Krueger and Duguay 1989; Schenk et al. 2021; Stepanek et al. 2020). Membership in a shooting club was declared three times, one male 17-year-old subject with a BLL of 22.4 µg/L. Others reported dealing with lead during renovation activities or did not further specify the activity. Surprisingly, occupancy was negatively correlated with BLL. An explanation might be that comparatively new residents or parents of children and minors might not be fully aware of the local lead issue and thus might not be as prudent when preparing homegrown foods or cleaning the house from lead-containing dusts.

On the other hand, time spent in the garden was positively correlated with BLL and can be explained with a higher probability to inhale or unintentionally ingest lead-containing dusts.

### Measures to reduce BLL

To further reduce the lead body burden in the region, it is advisable to avoid unintended ingestion of lead-containing foods and dusts and of course direct ingestion of soils. Therefore, a focus on both personal hygiene, that is, regular handwashing especially before meals, and a regular cleaning routine of the household should be implemented. Since children do lack permanent awareness, this represents a task that must be done by the caregivers. This is especially the case for the youngest ones when playing in the garden. Hand-to-mouth contact while playing in the garden and thus the threat of ingesting larger amounts of lead should be avoided as far as possible. Homegrown fruits and vegetables should be carefully washed or pealed and, depending on the ability to accumulate lead, be avoided completely.

However, in the context of severe lead intake, the administration of chelating agents is still regularly discussed as a possibility to treat heavy metal poisoning (Stepanek et al. 2020). This is not a recommended measure, as it mobilizes a large number of other heavy metals including essential trace elements. BLLs like the ones observed in this study do not give any reason to take such drastic measures. An exposure reduction is entirely sufficient. Some researches discuss a potential protective effect of selenium and zinc related to an elevated heavy metal burden, but from our point of view, reduction measures should be given preference (Rahman et al. 2019).

Soil analysis for lead content can help to identify extreme lead concentrations giving way for further protective measures like soil exchange or soil covering where deemed appropriate. Finally, lifestyle-related lead exposure due to renovation works or membership in a gun club should be reduced to a minimum.

Protective measures like an information campaign and analysis and exchange of kindergarten playground soil had been initiated already in the region. A permanent health desk enabling citizens to determine their BLL and receive advice is scheduled. Unfortunately, due to the catastrophic flooding events in July 2021, measures like soil exchange and covering of lead-rich ground layers were foiled to an unknown extent because of mud and river sediments precipitating all over the region.

## Conclusion

It was demonstrated that children and minors of both sexes in the region of Mechernich and Kall are more exposed to lead than the corresponding German general population. Thirty-two (17.6%) of the children and minors investigated had BLLs above the according RVs, instead of 5% expected from the general population. The absolute exceedance of the according RVs derived from the 95th percentile was about 10 to 15 µg/L with a maximum measured BLL of 44.0 µg/L. Measures to reduce the lead exposure should be initiated or enhanced.

As extracted from the data, apart from lifestyle habits, direct or indirect contact with soils via hand-to-mouth contact in the garden, a prolonged garden time, and higher frequency of consumption to homegrown products contributed to the subjects BLL.

Exchange or covering of soil in regions with known high lead soil content like in Strempt or near the river Bleibach should be considered; further, reduced consumption or more intense washing of homegrown products; more focus on personal hygiene, that is, for example, more careful handwashing before eating; and a general awareness of the topic of lead-contaminated soil might help, to reduce the lead burden in children and minors in the region.

However, it should be pointed out that about ten-fold higher mean BLLs predominated in the 1970s; that way, the actual observed exceedances do not pose a reason neither for an all-clear signal nor for sensationalizing of the results. A steady path and effort to reduce the lead burden in the region should be followed consequently since up to date no BLL that can be considered as safe exists.

The flooding event taking place shortly after this study is assumed to have foiled measures already undertaken. Therefore reconstruction, cleanup, and remediation measures of the flooded areas should comprise lead reducing measures wherever possible. Awareness of a low-key risk dwelling in the area should be maintained and extended in the residents. Furthermore, on top of the repeated measurements of BLL of children and minors that exceeded the according RV, neurological and cognitive test might give insight in potential adverse effects occurring due to lead exposure.

## Supplementary Information

Below is the link to the electronic supplementary material.

Supplementary file1 (XLSX 29 KB)

Supplementary file2 (DOCX 21 KB)

## Data Availability

Data and materials are provided in a supplementary file.
