# Supplementary material for: Blood lead monitoring in a former mining area in Euskirchen, Germany: results of a representative random sample in 3- to 17-year-old children and minors
Source: Environ Sci Pollut Res Int. 2022 Oct 20;30(8):20995–1009. doi: 10.1007/s11356-022-23632-2 (PMC9584279; doi:10.1007/s11356-022-23632-2)
Supplement: Supplementary file 2 — Supplementary file2 (DOCX 21 KB) [file 11356_2022_23632_MOESM2_ESM.docx]

Tab. S1 Target composition of the sample collective and realised sample collective of selected participants.

|  | Target | Actual | Target | Actual | Target | Actual | Target | Actual |  |
| --- | --- | --- | --- | --- | --- | --- | --- | --- | --- |
|  | **Kall** | **Kall** | **Mechernich** | **Mechernich** | **Both** | **Both** |  |  |  |
| **Age [a]** | **N female / male** | **N female / male** | **N female / male** | **N female / male** | **N female / male** | **N female / male** | **N sum** | **N sum** | **Actual %** |
| **3** | 2 / 2 | 0 / 2 | 6 / 4 | 3 / 1 | 8 / 6 | 3 / 3 | 14 | 6 | 43 |
| **4** | 2 / 2 | 2 / 2 | 4 / 6 | 2 / 7 | 6 / 8 | 4 / 9 | 14 | 13 | 93 |
| **5** | 2 / 2 | 1 / 1 | 6 / 4 | 5 / 5 | 8 / 6 | 6 / 6 | 14 | 12 | 86 |
| **6** | 2 / 2 | 3 / 1 | 4 / 6 | 4 / 6 | 6 / 8 | 7 / 7 | 14 | 14 | 100 |
| **7** | 2 / 2 | 1 / 1 | 4 / 4 | 2 / 5 | 6 / 6 | 3 / 6 | 12 | 9 | 75 |
| **8** | 2 / 2 | 2 / 1 | 4 / 4 | 4 / 4 | 6 / 6 | 6 / 5 | 12 | 11 | 92 |
| **9** | 2 / 2 | 0 / 3 | 4 / 4 | 6 / 3 | 6 / 6 | 6 / 6 | 12 | 12 | 100 |
| **10** | 2 / 2 | 2 / 1 | 4 / 6 | 3 / 6 | 6 / 8 | 5 / 7 | 14 | 12 | 86 |
| **11** | 2 / 2 | 2 / 3 | 4 / 6 | 3 / 5 | 6 / 8 | 5 / 8 | 14 | 13 | 93 |
| **12** | 2 / 2 | 2 / 1 | 4 / 4 | 6 / 4 | 6 / 6 | 8 / 5 | 12 | 13 | 108 |
| **13** | 2 / 2 | 2 / 2 | 6 / 6 | 4 / 6 | 8 / 8 | 6 / 8 | 16 | 14 | 88 |
| **14** | 2 / 2 | 2 / 0 | 4 / 6 | 6 / 6 | 6 / 8 | 8 / 6 | 14 | 14 | 100 |
| **15** | 2 / 2 | 3 / 1 | 6 / 4 | 4 / 5 | 8 / 6 | 7 / 6 | 14 | 13 | 93 |
| **16** | 2 / 2 | 1 / 1 | 4 / 6 | 2 / 5 | 6 / 8 | 3 / 6 | 14 | 9 | 64 |
| **17** | 2 / 2 | 1 / 3 | 6 / 6 | 6 / 7 | 8 / 8 | 7 / 10 | 16 | 17 | 106 |
| **Total** | 30 / 30 | 24 / 23 | 70 / 76 | 60 / 75 | 100 / 106 | 84 / 98 | **206** | **182** | **88** |

Tab. S2 Comparison of the results of this study with the results of Vogel et al. (2021)

|  | GerES V | Euskirchen | GerES V | Euskirchen |
| --- | --- | --- | --- | --- |
|  |  | **Lead [µg/L]** |  |  |
| **Sex / Age** | **Mean** | **Mean** | **95. Perc.** | **95. Perc.** |
| Male | 10.9 | 13.4 | 20.9 | 31.4 |
| Female | 10.4 | 10.5 | 19.2 | 22.9 |
| All 3-5 years | 11.5 | 14.6 | 23.1 | 35.3 |
| All 6-10 years | 11.7 | 13.2 | 20.3 | 35.1 |
| All 11-13 years | 9.2 | 9.6 | 17.5 | 17.2 |
| All 14-17 years | 9.8 | 11.1 | 15.6 | 27.6 |
| Total | 10.6 | 12.1 | 19.9 | 28.5 |

**General**

What is the sex of your child?

□ Male

□ Female

□ Divers

What is your child's height? ________(cm) □ Unknown

What is your child's weight? ________(kg) □ Unknown

Living situation

In which country was the child born?

□ Germany

□ Other country _____________________

Does your child change homes for reasons of changing custody so that it lives in Mechernich/Kall less than half the time?

□ No

□ Yes

Has your child taken a vacation outside of Mechernich/Kall in the last 6 months?

□ No

□ Yes

If yes, approximately when and for how long? ____________________________________

Resident at current address for ____ years.

Year of construction of the house

□ Before 1973

□ 1973-2000

□ after 2000

Is there a garden at the current residence/apartment that is used?

□ No

□ Yes

What is your child's usual garden time in hours per week? _________

How much time in hours per week does your child spend at playgrounds, soccer fields, etc.?

_______________

Does your child play in the garden and like to put objects (e.g., soil, rocks, ...) in its mouth?

□ No

□ Yes

Eating habits

How often does your child eat fruits or vegetables grown in your own garden?

□ Daily

□ Several times a week

□ Once a week

□ Less often

□ Never

Which________________________________________________________________

Do you have your own drinking water well?

□ Yes

□ No

Do you eat offal?

□ No

□ Yes. Which ones? __________________________________________

More often than 1x/week?

□ No

□ Yes, ___x/week

**Stimulants - smoking/passive smoking.**

Parent/other

Do you/your partner/other persons smoke or "vape/smoke e-cigarettes" in the home in the presence of your child?

□ No

□ Yes

□ Daily

□ Several times a week

□ Once a week

□ Less often

Child:

Does your child smoke or "vape/smoke e-cigarettes"?

□ No

□ Yes

□ Daily

□ Several times a week

Once a week

□ Less often

What smoking products are used?

Parent/Other: Child:

Type Amount x/daily Type Amount x/daily

□ Cigarettes ____ □ Cigarettes ____

□ E-cigarettes ____ □ E-cigarettes ____

□ Cigars ____ □ Cigars ____

□ Pipes ____ □ Pipes ____

□ Roll-your-own cigarettes ____ □ Roll-your-own cigarettes ____

□ E-shisha ____ □ E-shisha ____

□ hookah water pipe ____ □ hookah water pipe ____

□ Other: ______________ ____ □ Other: _______________ ____

**Other / Lifestyle**

Does your child privately handle lead?

□ Shooting club

□ Handling paint, for example, during renovation work;

If known Product _______________________________

□ Other: ______________________________

**Pre-existing conditions / medication**

Has your child ever been diagnosed with anemia requiring treatment?

□ No

□ Yes, most recently at __________________

Does your child have any known pre-existing conditions?

□ No

□ Yes, the following _______________

Is your child currently taking any medications?

□ No

□ Yes

□ Prescribed by doctor

□ Purchased at pharmacy without prescription.

□ Alternative remedy (homeopathic - ayurvedic)

□ The following medicines: ________

Has your child contracted SARS-CoV-2 or other infections in the past 3 months?

□ No

□ Yes

If Yes:

Was it in quarantine as a result and unable to be outside the apartment/house during that time?

□ No

□ Yes

□ For how long? ____(days)
